# Simulation of the Irradiation Cascade Effect of 6H-SiC Based on Molecular Dynamics Principles

**DOI:** 10.3390/mi14020455

**Published:** 2023-02-15

**Authors:** Yaolin Chen, Hongxia Liu, Tianzhi Gao, Hao Wei

**Affiliations:** Key Laboratory for Wide Band Gap Semiconductor Materials and Devices of Education, School of Microelectronics, Xidian University, Xi’an 710071, China

**Keywords:** 6H-SiC, molecular dynamics, irradiation cascade effect, PKA, SKA

## Abstract

When semiconductor materials are exposed to radiation fields, cascade collision effects may form between the radiation particles in the radiation field and the lattice atoms in the target material, creating irradiation defects that can lead to degradation or failure of the performance of the device. In fact, 6H-SiC is one of the typical materials for third-generation broadband semiconductors and has been widely used in many areas of intense radiation, such as deep space exploration. In this paper, the irradiation cascade effect between irradiated particles of different energies in the radiation and lattice atoms in 6H-SiC target materials is simulated based on the molecular dynamics analysis method, and images of the microscopic trajectory evolution of PKA and SKA are obtained. The recombination rates of the Frenkel pairs were calculated at PKA energies of 1 keV, 2 keV, 5 keV, and 10 keV. The relationship between the number of defects, the spatial distribution pattern of defects, and the clustering of defects in the irradiation cascade effect of 6H-SiC materials with time and the energy of PKA are investigated. The results show that the clusters are dominated by vacant clusters and are mainly distributed near the trajectories of the SKA. The number and size of vacant clusters, the number of Frenkel pairs, and the intensity of cascade collisions of SKAs are positively correlated with the magnitude of the energy of the PKA. The recombination rate of Frenkel pairs is negatively correlated with the magnitude of the energy of PKA.

## 1. Introduction

With the rapid development of aerospace technology, extreme environments (e.g., strong radiation fields) are placing new demands on the radiation resistance of semiconductor materials. In the third generation of semiconductors, devices prepared from the semiconductor material of SiC have the advantages of high temperature and high voltage resistance, high conductivity, and fast switching speeds. Different structures of SiC materials have different physical and electrical properties. In terms of silicon carbide crystalline materials, 3C-SiC materials are mainly used in photovoltaic applications, such as solar cells [1,2]. Indeed, 4H-SiC and 6H-SiC are most widely used in the semiconductor field, where 4H-SiC is mainly used for the preparation of high-frequency, high-temperature, and high-power devices [3,4,5]. In contrast, 6H-SiC materials are mainly used for the production of power devices in the field of optoelectronics, such as photoconductive detectors or substrate materials for LED devices [6,7]. From the current research progress, there have been many reports on the molecular dynamics of point defects arising from irradiation displacement damage in SiC, but most of them focus on 3C-SiC and 4H-SiC crystals [8,9,10,11,12], and 6H-SiC is often neglected, therefore the object of this paper is 6H-SiC. In fact, 6H-SiC crystals are a wide band gap material, meaning that the electronic devices made can work more reliably in harsh environments and can be widely used in aerospace, nuclear technology, and other high-temperature, high-radiation scenarios. In the study of phase transformation and amorphization of SiC, Aikaterini Flessa, M.Gloginjić, and others have done many experiments on the effect of irradiation cascade of 6H-SiC, and most of the researchers have studied the amorphization of 6H-SiC by high-energy, fast heavy ion injection using Raman spectroscopy and transmission electron microscopy methods [13,14,15,16]. K.Kamalakkannan et al., studied the induction behaviour and recrystallisation of 6H-SiC at different depths by low-energy ion injection [17,18]. In terms of simulations, J.B.Casady et al., studied the displacement threshold energy of SiC lattice atoms [19,20,21]. Liu C et al. studied the 3C-SiC irradiation cascade effect and summarised the distribution law of defect clusters [22]. Liao W et al. studied the effect of 6H-SiC at different irradiation energies on the irradiation cascade effect. However, there is a significant discrepancy between the results of Liao W et al. and the theoretically calculated values of the Kinchin–Pease model [23].

In order to understand the state of point defects arising from the irradiation cascade effect of 6H-SiC, this paper uses molecular dynamics (MD) analysis to simulate the evolution of point defects and clusters in Si–PKA (Primary Knock-on Atoms) incident targets. In recent years, the MD technique has been widely used in atomic-scale simulations as its simulation time step can be set to the fs level and therefore details of the irradiation cascade effect can be evolved at the atomic level. The number of defects, the recombination rate of defects, the generation of clusters, and the spatial distribution pattern of point defects can be obtained by applying the MD technique at different energies of the PKA.

## 2. Modelling of 6H-SiC and Experimental Design

**Figure 1:**
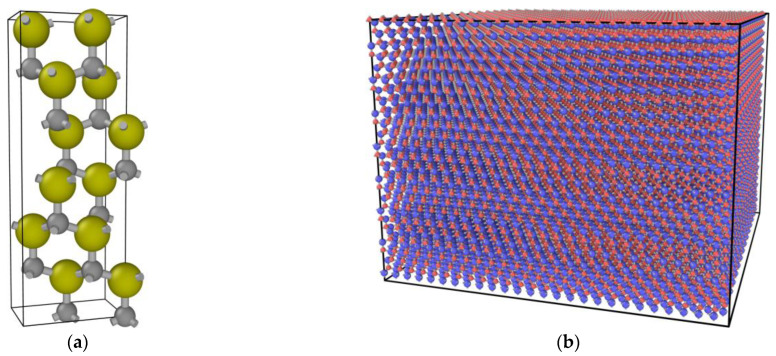
(**a**) The cell model of 6H-SiC; (**b**) The supercell model of 6H-SiC.

In this study, the Large-scale Atomic\Molecular Massive Parallel Simulator (LAMMPS) was used for modelling and irradiation simulations experiments on the 6H-SiC target material. The Open Visualization Tool (OVITO) was used for defect identification and cluster analysis. The 6H-SiC crystal is a hexagonal crystal structure with a lattice constant of a=b=0.3081 nm, c=1.5117 nm, and the lattice constants in the orthogonal coordinate system are $a^{a}=0.3073$ nm, $b^{b}=0.5322$ nm, $c^{c}=1.5117$ nm. The 6H-SiC crystal cell and supercell established in the orthogonal coordinate system are shown in Figure 1a and Figure 1b, respectively.

**Figure 2:**
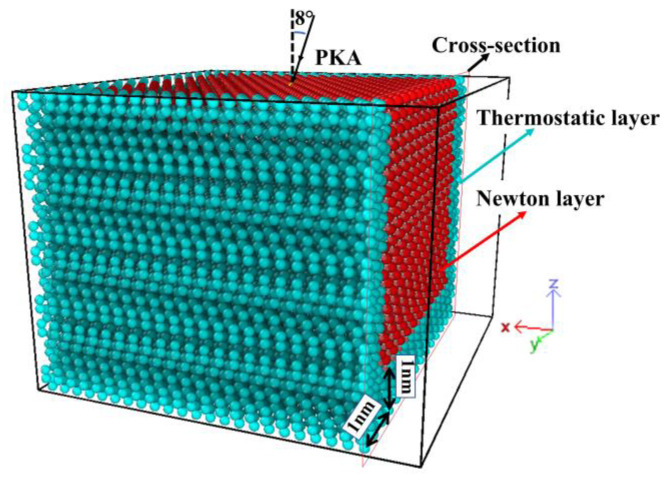
Simulation model.

Figure 2 shows a model of a supercell with full periodic boundary conditions. A silicon atom on the upper surface of the supercell is selected as the Primary Knock-on Atom (PKA). In addition to the top of the (001) face of the model, a 1 nm thick thermostatic layer is set at the boundary of the model, which avoids artificially reducing the energy of the system when the PKA is incident on the target material. The central region of the model is the Newtonian layer, which is the main cascade collision region.

**Figure 3:**
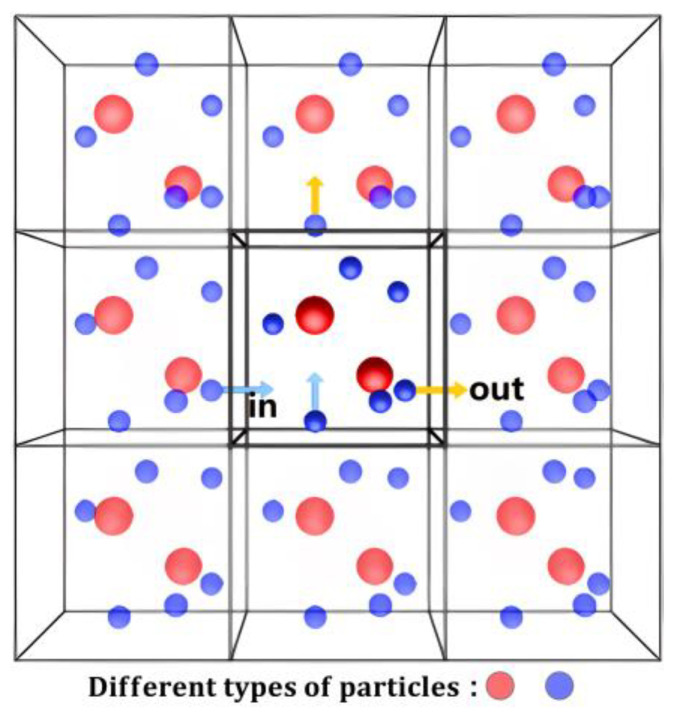
Schematic representation of the concept of periodic boundary conditions.

In Figure 3, as a particle in the simulation cell moves out of the system from one boundary, an identical particle enters the simulation cell from the opposite boundary to keep the total energy of the simulation cell and the number of particles constant. SiC devices are typically available in the size range of um or even mm. The computer resources and time consumed for molecular dynamics simulations of whole natural systems would be enormous due to the limitations of computer computing resources. Periodic Boundary Conditions (PBC) are a set of boundary conditions that typically approach large (infinite) systems by using a small fraction of what is called a cell. PBC allows for a more comprehensive calculation of the interatomic forces within the material, effectively eliminating the influence of boundary effects on the simulation results [24]. Since the molar mass and atomic radius of Si atoms are larger than those of C atoms, Si atoms have a higher probability of colliding with atoms in the target material, and the corresponding phenomenon of cascade collision effects will be more pronounced. Therefore, the choice of Si as PKA in this model is effective in observing the evolution of defects in the target material compared to C. In addition, PKA is not incident along the negative direction of the *Z*-axis to avoid trenching effects [25,26,27].

**Figure 4:**
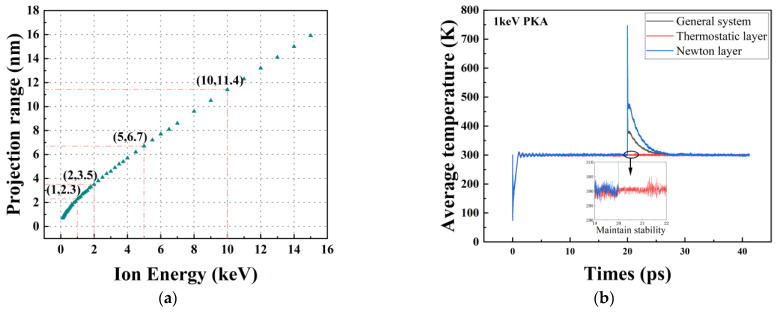
(**a**) Projected range of incident protons as a function of proton energy calculated by SRIM software; (**b**) Average temperature versus time curve for the simulated system at 1 keV PKA.

Figure 4a shows that the projection range of PKA obtained by SRIM [28] determines the lower limit of the thickness of the Newtonian layer. The SRIM simulation software does not take into account thermal effects and the simulated irradiation experiments performed in the SRIM software are performed in a 0K environment and cannot be artificially altered. Both temperature and angle of incidence affect the size of the Newtonian layer, so the model monitors the mean output temperature of the thermostatic layer to ensure that most or all of the cascade collisions occur in the Newtonian layer. In addition, the stability of the mean output temperature of the thermostatic layer determines whether the model is appropriately sized. When the size of the Newtonian layer is too small, cascade collisions are likely to occur in the thermostatic layer, leading to temperature fluctuations in the thermostatic layer. Figure 4b shows the average output temperature in different regions when 1 keV PKA is incident on the 6H-SiC target material. When the Newton layer is properly sized, natural heat transfer occurs in the Newton layer, transferring heat to the thermostatic layer. As a result, the average output temperature of the thermostatic layer does not fluctuate drastically at the start of the collision. The results show that the dimensions of the simulated system are suitable when the dimensions of the simulation system are length:width:thickness = 3:3:4, and the specific experimental parameters are designed as shown in Table 1.

For the simulation method, the interatomic interaction forces are described by the Tersoff/ZBL [29,30,31] potential function, with the Tersoff potential function and the ZBL potential function describing the long-range and short-range interatomic interaction forces, respectively. The simulated system uses the temperature control by velocity rescaling (temp/rescale) method. It is based on the principle that when the temperature exceeds the set temperature, the temperature is adjusted by scaling the atomic velocity to reach the set value. The system is relaxed for 20 ps using the NVT ensemble to bring the system into equilibrium. The PKA will be incident at an angle of 8° along the negative direction of the *Z*-axis at a certain velocity. In the irradiation simulation experiments, the NVE ensemble will be used with a simulation time of 21.2 ps and the simulation parameters were set in Table 2.

The expression between the energy and velocity of PKA is given by Equation (1).

(1) $$E=\frac{1}{2}mv^{2}$$

E is the energy, m is the atomic mass and v is the atomic velocity in J, kg, m/s respectively. For the simulated system in this thesis, the atomic velocity is given in $Å\cdot {\mathrm{ps}}^{-1}$ and is converted to the form of Equation (2).

(2) $$v^{`}=\sqrt{\frac{2*1.6*10^{-16}*E^{`}}{1.66*10^{-26}*m^{`}}}$$

$E^{`}$ is the irradiation energy, $m^{`}$ is the molar mass of the atom and $v^{`}$ is the atomic velocity in eV, g/mol, $Å\cdot {\mathrm{ps}}^{-1}$ respectively.

The number of vacancy-interstitial pairs (Frenkel pairs) is an important indicator of the extent of radiation damage when an irradiation cascade effect occurs in the target material. Norgett et al. [32] proposed Equation (3) to approximate the number of Frenkel pairs produced by the cascade collision effect, based on the expression given by Kinchin and Pease [33], where $N_{d}$ is the number of Frenkel pairs; $E_{v}$ is the energy of the incident particle; and $E_{d}$ is the off-site threshold energy of the target atom.

(3) $$N_{d}=\{\begin{array}{c} \;\;\;\;\;0\;\;\;\;\;\;\;\;\;\;\;,\;\;E_{v}<E_{d}\;\;\;\;\;\;\;\;\;\;\;\;\;\\ \;\;\;\;\;1\;\;\;\;\;\;\;\;\;\;\;,\;\;E_{d}\leq E_{v}<\frac{2*E_{d}}{0.8}\\ \;\;\;\;\;\frac{0.8*E_{v}}{2*E_{d}}\;\;\;\;\;\;\;\;,\;\;E_{v}\geq \frac{2*E_{d}}{0.8}\;\;\;\;\;\;\;\;\;\;\;\;\;\;\;\; \end{array}$$

**Figure 5:**
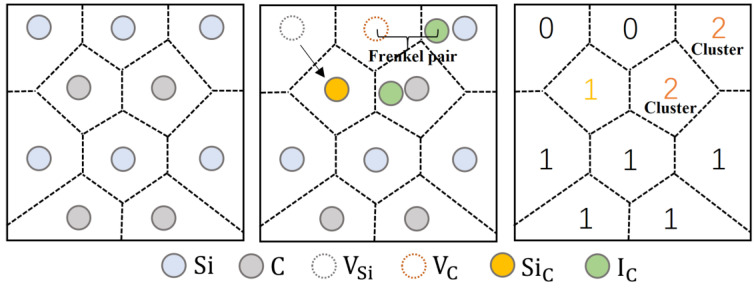
Defect identification method.

Figure 5 shows the Wiger-Seitz defect identification method, calculated separately for Frenkel pairs (one vacant and one interstitial atom), silicon vacant atoms ($V_{\mathrm{Si}}$), carbon vacant atoms ($V_{C}$), silicon interstitial atoms ($I_{\mathrm{Si}}$), carbon interstitial atoms ($I_{C}$), silicon anti-substitial atoms (${\mathrm{Si}}_{C}$) and carbon anti-substitial atoms ($C_{\mathrm{Si}}$), respectively, where the cluster formation has a search radius of 0.22 nm [22,34,35].

## 3. Results and Discussion

### 3.1. Evolution of PKA Energy and Frenkel Pairs over Time

This section compares the obtained defect generation efficiency with the available literature data by analysing the difference between the number of defects in the simulated system and the theoretical values calculated from the modified Kinchin–Pease equation. It was used to assess the accuracy of the model in this paper. The K-P equation is commonly used to describe the relationship between the number of defects produced by the irradiation cascade effect and the incident energy of the PKA ($E_{PKA}$). In the case of 6H-SiC targets material, the K-P equation can be expressed in the form of Equation (4).

(4) $$N_{d}=\frac{0.4\cdot E_{PKA}}{\frac{1}{a_{1}+a_{2}}(a_{1}\cdot E_{d1}+a_{2}\cdot E_{d2})}$$

where $N_{d}$ is the predicted number of defect pairs and $E_{PKA}$ is the energy of PKA (eV). $E_{d1}$ and $E_{d2}$ are the off-site threshold energies (eV) of the atoms in the target material, 20 eV and 35 eV for carbon and silicon atoms respectively [20]. $a_{1}:a_{2}=r_{1}:r_{2}$, where $r_{1}$ is the atomic radius of the C atom (0.091 nm); $r_{2}$ is the atomic radius of the silicon atom (0.25 nm).

**Figure 6:**
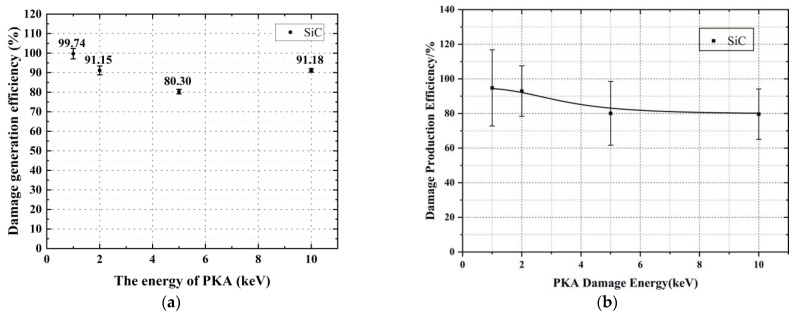
Shows a plot of the defect generation efficiency. (**a**) Results of this model; (**b**) Results from the literature [23].

In Figure 6, the damage generation efficiency represents the ratio of the number of simulated Frenkel pairs to the theoretically calculated value of Equation (4). The results in Figure 6a show that the majority of damage generation efficiencies in this paper are greater than 90%, accurately reflecting the generation of Frenkel defects in 6H-SiC crystals. Figure 6b shows the generation efficiency of the Frenkel pair from the literature [23]. The crystal material used in this model is also 6H-SiC. The comparison of the data shows that the damage generation efficiency in the literature [23] is lower than that of the present model, implying that the results obtained through the present model are closer to the theoretical values. In addition, the length of the error bars for damage generation efficiency in the literature [23] is also longer than the length of the error bars for damage generation efficiency in the present model, which means that the present model is more stable and reliable.

At 20 ps, the PKA starts bombarding the 6H-SiC target material. The PKA collides with the lattice atoms in the target material and the energy of the PKA is transferred to the lattice atoms. There are two phenomena with lattice atoms: (1) The energy gained reaches the delocalisation threshold and interstitial atoms are formed; (2) The energy absorbed is less than the delocalisation threshold energy and thermal vibrations are produced in the 6H-SiC target material. In order to study the relationship between the PKA energy absorbed by the system and the Frenkel pairs, it is necessary to know the evolution of the PKA energy and the number of Frenkel pairs with time.

**Figure 7:**
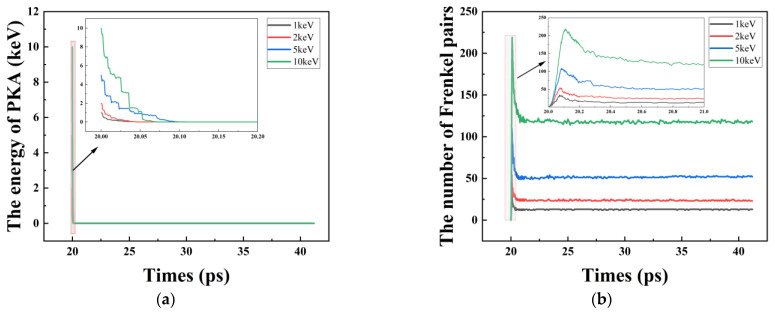
(**a**) The energy of PKA versus time; (**b**) Number of Frenkel pairs versus time.

In Figure 7a, most of the energy of the PKA is consumed between 20 and 20.1 ps. Between 20.1 and 20.2 ps, the PKA energy loss is exhausted, while the higher PKA energy loss takes longer to be exhausted. In Figure 7b, the number of Frenkel pairs peak near the 20.1s moment, and the higher the energy of the PKA, the later the peak appears. In the phase after 20.1 ps, the final number of Frenkel pairs decreases as the target material undergoes a high-temperature annealing phase. After the 21 ps moment, it stabilizes, with a trend consistent with that of ZHOU Y et al. [36]. In time, the peak of Frenkel pairs appears at the same time as the PKA energy is almost depleted, so the number of Frenkel pairs corresponds to the PKA energy as follows: (1) The number of Frenkel pairs increases with the loss of PKA energy. (2) When the PKA kinetic energy is exhausted, the number of Frenkel pairs begins to decrease. (3) As the system temperature slowly recovers to 300 K, the number of Frenkel pairs stabilises.

**Figure 8:**
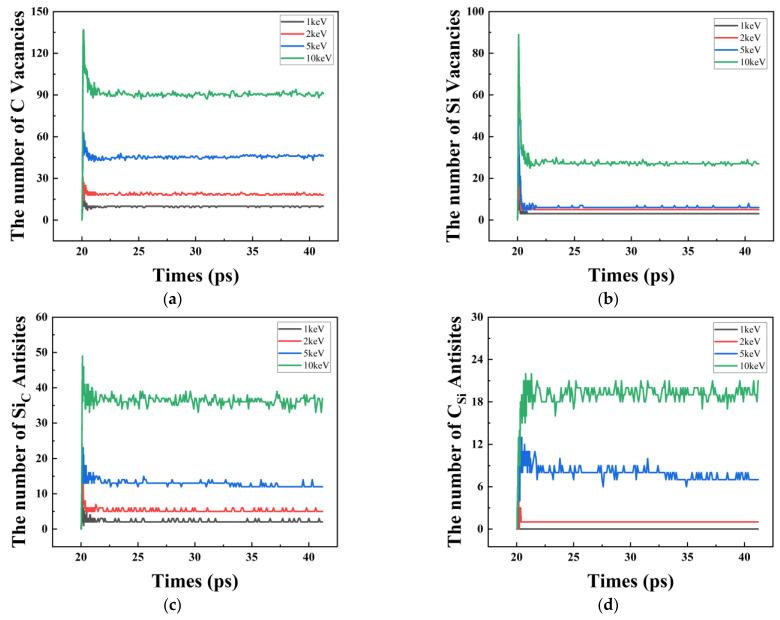
(**a**) Number of $V_{c}$ versus time; (**b**) Number of $V_{\mathrm{Si}}$ versus time; (**c**) Number of ${\mathrm{Si}}_{C}$ versus time; (**d**) Number of $C_{\mathrm{Si}}$ versus time.

In Figure 8a,b depict the evolution of different types of point defects with time for different PKA energy conditions, with a similar pattern of variation to the number of Frenkel pairs. In the stablilisation phase of the simulation system, the vacancy defects are mainly $V_{C}$, the reason being that the relative molar mass of the C atom is smaller than that of the Si atom and the threshold energy required for collisional delocalisation is lower. Additionally in Figure 8c,d describe the number of ${\mathrm{Si}}_{C}$ and $C_{\mathrm{Si}}$ defects as a function of time. As the number of ${\mathrm{Si}}_{C}$ and $C_{\mathrm{Si}}$ increases rapidly, it means that $V_{C}$ and $V_{\mathrm{Si}}$ are consumed and the number decreases. The results show that the conversion of 6H-SiC from crystalline to amorphous is mainly influenced by two defect types, $V_{C}$ and ${\mathrm{Si}}_{C}$.

(5) $$\eta =1-\frac{N_{d}}{N_{max}}\times 100\%$$

where $N_{d}$ and $N_{max}$ are the number of Frenkel pairs and the maximum number of Frenkel defect pairs, respectively, during the simulation.

In addition, the relationship between the number of Frenkel pairs in the steady-state phase and the peak value of the Frenkel pairs is investigated, as the recombination rate of Frenkel pairs is an important indicator for assessing the radiation resistance of semiconductor materials. The definition of the Frenkel pairs recombination rate is given in Equation (5).

**Figure 9:**
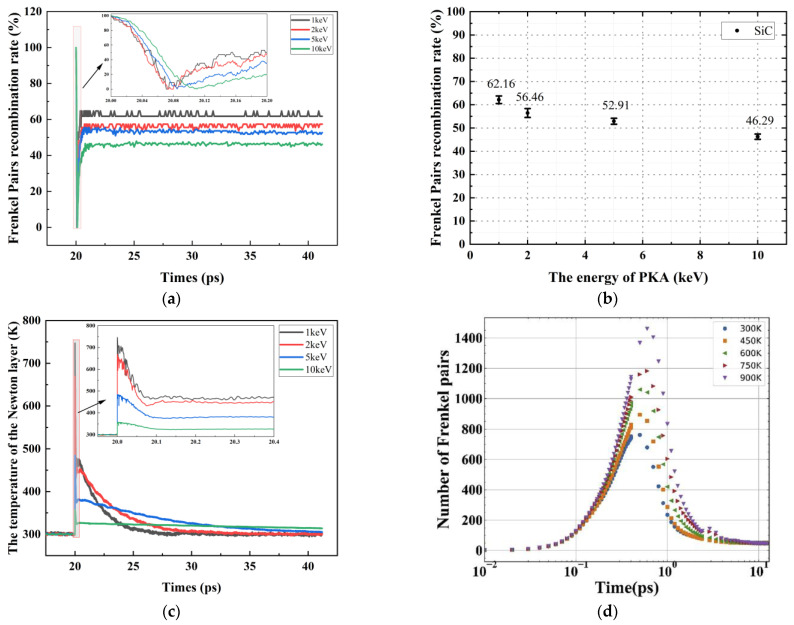
(**a**) Compounding rate of Frenkel pairs as a function of time; (**b**) Compounding rate of Frenkel pairs as a function of PKA energy; (**c**) Variation of Newtonian layer temperature as a function of time for different PKA energies; (**d**) Variation of the number of Frenkel pairs with time for different ambient temperature conditions [37]. The target material is GaN and the energy of the PKA is 10 keV.

Figure 9a shows that the moment of onset of the Frenkel pair peak is delayed backwards as the PKA energy increases. Thereafter, the recombination rate of the Frenkel pair is influenced by the thermal peak, which begins to rise and eventually remains stable. Figure 9b shows that the recombination rate of the Frenkel pair decreases as the PKA energy rises. Furthermore, Figure 9c shows that as the PKA energy increases, the irradiation cascade collisions result in more off-site atoms, thus reducing the thermal vibration phenomenon of the lattice atoms, the Newtonian layer temperature decreases and the weakening of the high-temperature annealing effect directly leads to a decrease in the recombination rate of the Frenkel pair.

Table 3 shows the physical parameters of the two semiconductor materials. GaN materials are widely used as one of the typical third-generation semiconductor materials. This paper compares the irradiation damage results of 6H-SiC and GaN materials and analyses the similarities and differences in the irradiation cascade effects of 6H-SiC and GaN target materials.

Figure 9d shows the results in the literature [37], where the simulated system uses a similar model to that used in this paper. The generation of Frenkel pairs of 6H-SiC and GaN is compared. The results show that (1) the GaN material has a higher peak for the Frenkel pair and the peak occurs later. This indicates that the cascade collision effect is stronger in GaN materials than in 6H-SiC, i.e., GaN materials are more sensitive to radiation. (2) After the high-temperature annealing stage, the number of surviving Frenkel pairs of GaN is less than that of SiC, and the composite rate of Frenkel pairs reaches over 90%, while the composite rate of Frenkel pairs of 6H-SiC in this paper is 46.29%. This means that GaN has a higher defect compounding efficiency than 6H-SiC and ends up with less radiation damage than 6H-SiC. Attention to the peak of the Frenkel pair is important for studying the resistance of materials to irradiation. The performance of the device is affected not only by the final radiation damage, but also by damage during the radiation process. The reason for this is that when a device in working condition is bombarded with radiation particles, the peak of the Frenkel pair is likely to cause extensive damage to the material, leading directly to the failure of the device.

### 3.2. Spatial Distribution of Defects

This subsection focuses on the spatial distribution of defects and their evolutionary patterns. In the irradiation cascade effect, irradiation defects may be distributed in sensitive areas of the device. Defects affect the device to different degrees at different moments or with different spatial distributions. For example, the device fails when the collision cascade effect is most pronounced and is not caused by a defect in the steady-state phase. Therefore, exploring the spatial distribution pattern of irradiation defects in 6H-SiC materials in time and space is an important reference for the improvement of the irradiation resistance of SiC-type semiconductor devices.

**Figure 10:**
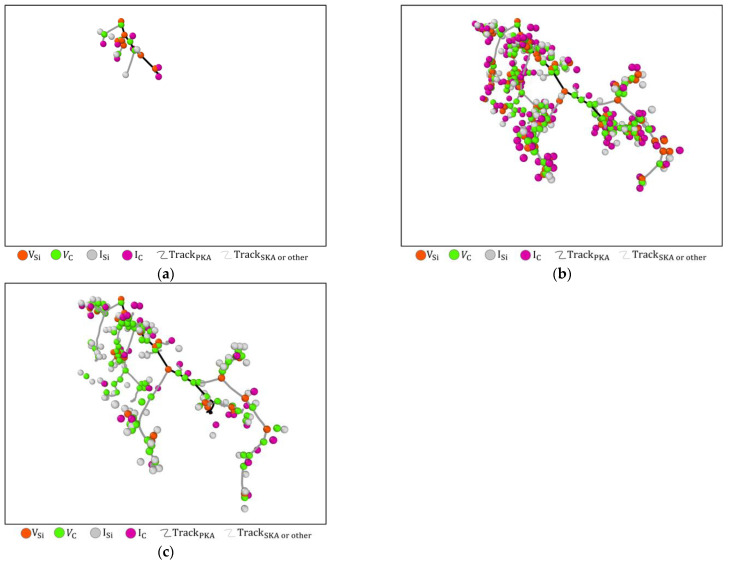
Spatial distribution of point defects produced by PKA at 10keV incident on a 6H-SiC target: (**a**) Early collision period; (**b**) Peak period; (**c**) Stable period.

In Figure 10, $V_{\mathrm{Si}}$ and $V_{C}$ are mainly distributed on the PKA and secondary knock-on atoms (SKA) trajectories, while $I_{\mathrm{Si}}$ and $I_{C}$ are mainly around $V_{\mathrm{Si}}$ and $V_{C}$. Meanwhile, SKA cascade collisions are more violent than PKA, with more and denser point defects distributed around SKA trajectories.

**Figure 11:**
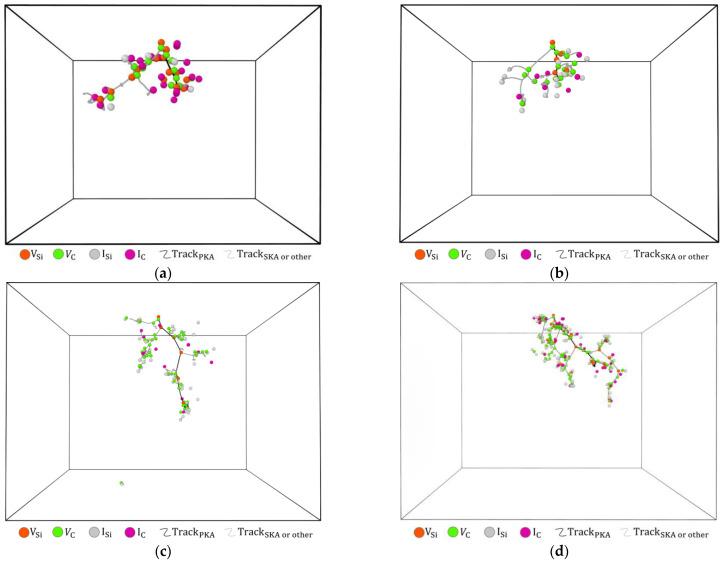
Spatial distribution of point defects in the stabilisation phase of 6H-SiC target materials at different energies of PKA. (**a**) 1 keV; (**b**) 2 keV; (**c**) 5 keV; (**d**) 10 keV.

In Figure 11, the point defects are distributed mainly along the PKA trajectories at 1 keV and 2 keV PKA energy conditions and are relatively discrete. At PKA energies of 5 keV and 10 keV, more SKA cascade collisions are produced and the proportion of point defects distributed around SKA trajectories has increased.

### 3.3. Cluster Analysis

The size of the defect clusters produced by irradiation cascade collisions affect the performance of semiconductor devices. Therefore, this section focuses on the analysis of the size of irradiated defect clusters formed in the peak moment and steady-state phase of the Frenkel pair for 6H-SiC target materials at different irradiation energies.

**Figure 12:**
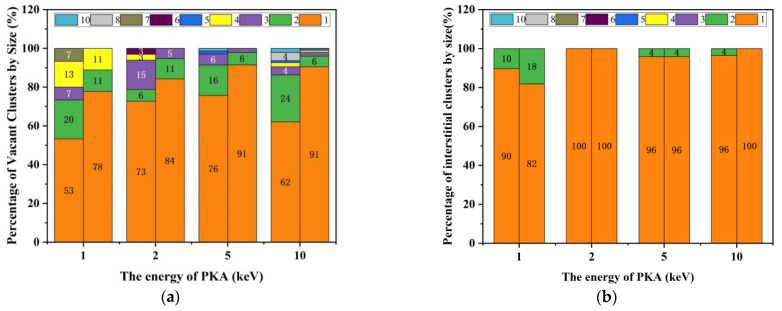
(**a**) Plot of the percentage stack of vacant clusters by size; (**b**) The percentage stack of interstitial clusters by size. Where the data on the left of each set of bar graphs corresponds to the peak and the data on the right to the steady-state phase.

In Figure 12, the proportion of large clusters (cluster size ≥ 2) at the Frenkel peak increases with increasing PKA energy and decreases in the stable phase, indicating that the defects produced by irradiation cascade collisions are predominantly in the form of point defects.

**Figure 13:**
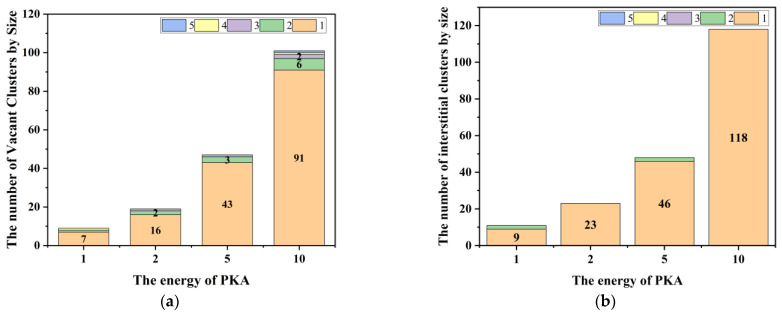
(**a**) Number of vacant clusters at different PKA energies; (**b**) Number of interstitial clusters at different PKA energies.

In Figure 13, the number of clusters (size ≥ 2) produced is greater with increasing PKA energy. Combined with the data in Figure 12, the results show that the proportion of clusters larger than 1 in size, although decreasing, increases with increasing PKA energy during irradiation cascade collisions, and that the larger-size clusters are dominated by vacant clusters.

**Figure 14:**
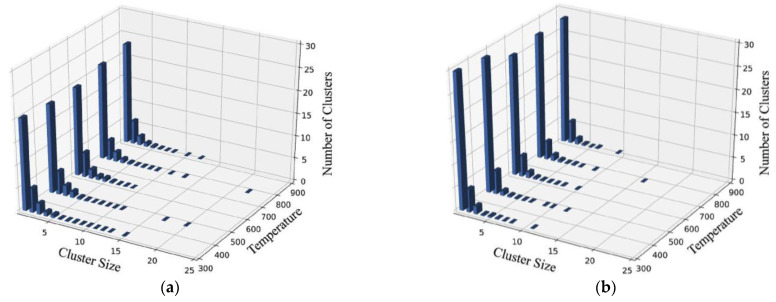
Clusters of different sizes versus ambient temperature [37]. (**a**) Vacancy clusters; (**b**) Gap atom clusters. The target material is GaN and the energy of PKA is 10 keV.

In Figure 14, the cluster generation of 6H-SiC and GaN was compared at 10 keV PKA and 300 K ambient temperature. The results are as follows: (1) 6H-SiC and GaN share a similar pattern in that clusters larger than 2 in size are mainly in the form of vacancy clusters; (2) In 6H-SiC target materials, vacancy defects are mainly point defects and the number of vacancy clusters is relatively small. Compared to 6H-SiC, the number of vacancy clusters in GaN targets is much higher and the resulting clusters are larger in size. This means that a more concentrated region of radiation damage is created in the Frenkel pairs of GaN materials with a quantity less than 6H-SiC.

## 4. Conclusions

The model in this paper can simulate the irradiation cascade effect in 6H-SiC target materials with relative accuracy. The results show that $V_{C}$ and ${\mathrm{Si}}_{C}$ are the main defect types responsible for the amorphization of 6H-SiC. In the stabilisation phase, the spatial distribution of point defects produced by PKA at 1 keV and 2 keV energies is discrete along the PKA trajectory, and the spatial distribution of point defects produced by PKA at 5 keV and 10 keV is more concentrated along the SKA trajectory. In the irradiation cascade effect of 6H-SiC, there is a good correlation between the defects and the energy of the PKA. The number of Frenkel pairs is linearly related to the energy magnitude of the PKA, and the Frenkel pairs recombination rate is negatively correlated with the energy magnitude of the PKA. Additionally, the number and size of vacancy clusters are positively correlated with the energy magnitude of PKA.

## Figures and Tables

**Table 1:** Design of parameters for simulation experiments.

Material	Energy of PKA	Model Size	Number of Atoms	$\mathrm{Velocity}\;(Å\cdot ps^{-1})$
6H-SiC *	1 keV	26a×15b×4c	37,440	829.7398136
	2 keV	33a×19b×5c	75,240	1173.429298
	5 keV	52a×30b×8c	299,520	1852.379976
	10 keV	92a×53b×14c	1,638,336	2191.765545

* $a^{a}=0.3073$ nm, $b^{b}=0.5322$ nm, $c^{c}=1.5117$ nm, Ambient temperature is 300 K.

**Table 2:** Parameters related to the simulation step.

Stage	Time Step	Number of Steps	Times
Relaxation phase	1 fs	20,000 steps	20 ps
Cascade collision phase	0.01 fs	20,000 steps	0.2 ps
	0.1 fs	10,000 steps	1 ps
Steady-state phase	1 fs	20,000 steps	20 ps

**Table 3:** Physical parameters of the material.

Item	6H-SiC	GaN
Band gap (300K) (eV)	3.02	3.42
Saturation electron transfer rate (cm/s)	2.0 × 10^7^	2.46 × 10^7^
Electron transfer rate (cm^2^)	400	1000
Thermal conductivity (W·cm^−1^·K^−1^)	4.5	2~3
Critical breakdown field strength (MV/cm)	2.4	3.3

## Data Availability

Not applicable.
